# Comprehensive Dissolution Study on Two Double Ce(IV) Phosphates with Evidence of Secondary CeO_2_ Nanoparticle Formation

**DOI:** 10.3390/molecules30102105

**Published:** 2025-05-09

**Authors:** Anastasiia L. Listova, Anastasiia S. Kuzenkova, Mikhail A. Gerasimov, Elizaveta S. Kulikova, Roman D. Svetogorov, Daniil A. Novichkov, Alexei A. Averin, Vasiliy O. Yapaskurt, Anna Yu. Romanchuk, Stepan N. Kalmykov, Tatiana V. Plakhova

**Affiliations:** 1 Faculty of Chemistry, Lomonosov Moscow State University, Leninskie Gory 1, 119991 Moscow, Russia; alvovna@yandex.ru (A.L.L.); kyznastya@mail.ru (A.S.K.); mishasmt@mail.ru (M.A.G.); rdsvetov@gmail.com (R.D.S.); danylo.novichkov@gmail.com (D.A.N.);; 2 National Research Centre Kurchatov Institute, Akademika Kurchatova pl. 1, 123182 Moscow, Russia; lizchkakul@mail.ru; 3 Frumkin Institute of Physical Chemistry and Electrochemistry, Russian Academy of Sciences, Leninsky Prospect 31 bld. 4, 119071 Moscow, Russia; alx.av@yandex.ru; 4 Faculty of Geology, Lomonosov Moscow State University, Leninskie Gory 1, 119991 Moscow, Russia; vyapaskurt@mail.ru

**Keywords:** Ce(IV) compounds, nanoparticles, phosphates, phase transformation, dissolution

## Abstract

Herein, we present a comprehensive study on the dissolution behaviour of two sodium–cerium(IV) phosphate phases synthesised hydrothermally from CeO_2_ nanoparticles: crystalline Na_2_Ce(PO_4_)_2_ and nanocrystalline NaCe_2_(PO_4_)_3_. For the first time, experimental dissolution data were obtained for both compounds over a wide pH range (1.5–10) under long-term equilibration. The crystalline phase undergoes pH-dependent transformation, including recrystallisation at a near-neutral pH and the formation of secondary CeO_2_ nanoparticles above pH 7. In contrast, the nanophase NaCe_2_(PO_4_)_3_ exhibits exceptional structural and chemical stability, showing no signs of recrystallisation, phase transformation, or CeO_2_ formation, even after extended ageing. The experimental results help refine the thermodynamic stability conditions for cerium phosphate and oxide phases, providing insights into the reversible transformation pathways between CeO_2_ and Ce(IV) phosphates as governed by pH.

## 1. Introduction

Inorganic phosphates represent a remarkable class of materials widely recognised for their rich structural chemistry, chemical diversity, and unique physicochemical properties [1,2,3,4,5,6]. Among their most valuable characteristics are exceptionally low solubility and high resistance to corrosion by geological fluids [7]. These features make phosphate-based materials particularly appealing for environmental remediation applications, notably as stable matrices for immobilising radioactive waste and selective adsorbents for contaminant removal from wastewater [8,9,10,11,12]. Phosphate-based ceramics such as britholites, monazite/brabantite solid solutions, and thorium phosphate diphosphate (β-TPD), as well as β-TPD/monazite composites, have been extensively studied for their exceptional chemical durability and potential application in actinide immobilisation. Consequently, understanding the dissolution mechanisms, long-term stability, and structural transformations of phosphate materials is essential for assessing their effectiveness in minimising the risks associated with radionuclide leaching into the environment.

Most available studies determined that lanthanide and actinide phosphates are considered soluble only under acidic conditions, reflecting strong binding between f-element cations and phosphate groups [7,13,14,15]. Tetravalent (e.g., Th, Pu) phosphates show significantly lower solubility than trivalent (e.g., La, Nd) ones. Reports indicate that the solubility product (log*K_sp_*) values for trivalent phosphates typically range from approximately −25 to −27, varying based on cation radii and trivalent phosphate structures (e.g., rhabdophane, xenotime) [7,16]. Thorium-based phosphates such as Th_2_(PO_4_)_2_(HPO_4_)·H_2_O or Pu_x/2_Th_2x/2_(PO_4_)_2_(HPO_4_) H_2_O exhibit extremely low solubility products (log*K_sp_*~−60) [7]. In many environments, phosphate precipitation can effectively limit actinide mobility [17,18].

Beyond the influence of solution acidity, several other factors critically impact materials’ solubility, including the crystal structure type, temperature, ageing time, or presence of complexing agents. Lanthanide phosphate solubility decreases with increasing temperature (up to 300 °C), a phenomenon referred to as retrograde solubility [19,20]. This behaviour is observed for certain rare-earth element phosphates and is attributed to the weak complexation of trivalent cation in solution, temperature-induced changes in material crystallinity, and modifications to hydration dynamics. Liu and Byrne also highlighted that freshly precipitated lanthanide phosphates are significantly more soluble than aged and well-crystallised counterparts, emphasising the importance of synthesis conditions and prolonged solution–solid interactions [16]. As a result of the dissolution process, the composition of the initial solid phase can change significantly, potentially altering the solubility-control phase and shifting the thermodynamic equilibrium of the system.

The present study focused on cerium phosphates, motivated by the unique redox chemistry of cerium, which can stably exist in both +3 and +4 oxidation states, in contrast to most other lanthanides that predominantly exhibit the +3 state. The Ce^3+^/Ce^4+^ redox couple enables redox-tuneable functionality, expanding the application range of cerium-based materials [21,22,23,24,25,26]. Although the dissolution behaviour of Ce(IV) oxide has been extensively studied in different media (including phosphate-rich media) [27,28,29,30,31,32], less attention has been devoted to cerium phosphates. Reliable thermodynamic data are primarily available for rhabdophane-type CePO_4_, while our knowledge of the solubility behaviour and stability of more complex Ce(IV) phosphate phases in different aqueous media remains limited. At the same time, Ce(IV) phosphates can exhibit greater structural diversity, as additional ions or functional groups (e.g., protons, hydroxyl groups, or other cations) are often incorporated to maintain charge neutrality. This can lead to various complex phosphates, such as hydrophosphates, hydroxyphosphates, and double phosphates [33,34,35,36,37]. Recently, Ce(PO_4_)(HPO_4_)_0.5_(H_2_O)_0.5_ was proposed as a promising sorbent for radionuclide removal [24]. Different Ce(IV) and Ce(III) phosphates are under consideration regarding use as components of sunscreens [25,38], further underscoring the importance of exploring the dissolution behaviour of Ce-containing phosphate systems. 

Thus, in this study, we systematically investigated the dissolution behaviour of two distinct sodium–cerium phosphate phases: the crystalline Na_2_Ce(PO_4_)_2_ and the nanostructured NaCe_2_(PO_4_)_3_. Both materials merit significant interest from fundamental and practical perspectives. The hydrated phosphate Na_1.97_Ce_1.03_(PO_4_)_2_·xH_2_O is a newly discovered crystalline material whose structure was resolved via X-ray diffraction and comprehensively characterised [34]. Our earlier research identified the formation of nanorod-structured NaCe_2_(PO_4_)_3_ during the prolonged transformation of CeO_2_ nanoparticles in the presence of phosphate species under environmentally relevant conditions [29]. We explored the dissolution process of these phosphates over an extensive pH range through under-saturation experiments, aiming to elucidate cerium concentrations in solution and associated structural transformations. Analyses of the aqueous phase were complemented by comprehensive analyses of the solid phases via synchrotron-based X-ray diffraction (XRD), Raman spectroscopy, and scanning electron microscopy (SEM), to identify the phases that could control solubility under varying environmental conditions.

## 2. Results

**Figure 1:**
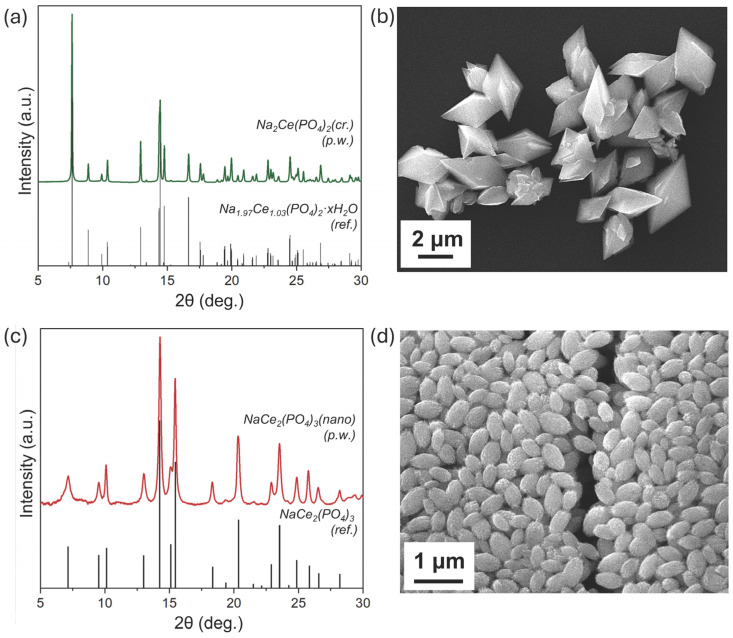
(**a**) XRD pattern and (**b**) SEM image of crystalline sodium–cerium phosphates (Na_2_Ce(PO_4_)_2_(cr.)) obtained after hydrothermal treatment of CeO_2_ nanoparticles in phosphate buffer at pH = 7.7. (**c**) XRD pattern and (**d**) SEM image of nanocrystalline sodium–cerium phosphates (NaCe_2_(PO_4_)_3_(nano)) obtained after hydrothermal treatment of CeO_2_ nanoparticles in phosphate buffer at pH = 4.4. ‘p.w.’ refers to experimental XRD patterns obtained at present work. The experimental XRD patterns are compared with literature-derived data for Na_1.97_Ce_1.03_(PO_4_)_2_·xH_2_O [34] and NaCe_2_(PO_4_)_3_ [29].

In the present study, sodium–cerium double phosphates were synthesised via a hydrothermal approach using CeO_2_ nanoparticles as the cerium precursor. The hydrothermal treatment was carried out in a 1 M sodium phosphate-buffer solution at two different pHs: 4.4 and 7.7. The XRD and SEM results for the obtained phosphate samples are presented in Figure 1, confirming the formation of double phosphate phases following the hydrothermal treatment of CeO_2_ nanoparticles. The structure and morphology of the resulting materials were found to be strongly dependent on the pH of the buffer solution.

The hydrothermal treatment of CeO_2_ nanoparticles at pH = 7.7 yielded a highly crystalline compound with well-defined, narrow diffraction peaks. The peak positions and relative intensities closely match those of a recently reported crystalline sodium–cerium double phosphate phase dominated by Ce(IV) [34] (Figure 1a). According to SEM analysis, the sodium–cerium double phosphate hydrothermally synthesised at pH = 7.7 exhibits well-developed rhombohedral crystals (Figure 1b). The average vertex-to-vertex diagonal of these crystals is approximately 3 μm. Energy-dispersive X-ray spectroscopy (EDX) performed during SEM analysis confirmed a Na/Ce atomic ratio of 2 (Table S1). Thermogravimetric analysis (TGA) showed negligible mass loss (<1.5%), suggesting that the compound is effectively unhydrated (Figure S1). Therefore, this phase is hereafter referred to as Na_2_Ce(PO_4_)_2_(cr.).

Due to the variability in phosphate polyhedron arrangements and the partial oxidation of Ce^3+^ to Ce^4+^, relatively few tetravalent cerium phosphate structures have been refined to date [33,35,39,40]. The structure of Na_1.97_Ce_1.03_(PO_4_)_2_·xH_2_O is one of those that have been thoroughly characterised. According to Baranchikov et al. [34], this sodium–cerium phosphate adopts a tunnel-type crystal structure (space group °*P*2_1_/*c*) featuring mixed oxidation states of cerium. The structure consists of CeO_8_ tetragonal antiprisms interconnected with PO_4_ tetrahedra.

Following the treatment of CeO_2_ nanoparticles in phosphate buffer at pH = 4.4, a sodium–cerium double phosphate also formed, but with a distinct structure compared to Na_2_Ce(PO_4_)_2_(cr.). The diffraction peaks of this sample are noticeably broader. The average crystallite size in this sample, calculated from the most intensive peaks’ full width at half maximum (FWHM) using the Scherrer equation, is 23 ± 2 nm, confirming its nanocrystalline nature (Figure 1c). SEM images reveal that the product consists of spindle-like aggregates composed of nanorods, clearly showing the anisotropic morphology of the synthesised phosphate phase (Figure 1d). These aggregates are approximately 0.3 μm in width and up to 0.5 μm in length.

The diffraction pattern and unique morphology of this phosphate phase are consistent with NaCe_2_(PO_4_)_3_, which we previously discovered (hereafter referred to as NaCe_2_(PO_4_)_3_(nano)). In our earlier work, this nano sodium–cerium phosphate was identified as a product of the long-term phosphate-induced transformation of CeO_2_ nanoparticles under mildly acidic conditions (pH ~4) at 25 °C over up to four years. Due to its strong anisotropy and nanoscale nature, the refinement of its crystal structure from XRD data proved difficult; however, the material was extensively characterised using other techniques. X-ray absorption spectroscopy confirmed that cerium predominantly exists in the Ce(IV) oxidation state. Pair distribution function analysis of XRD data further revealed that the local crystal structure is analogous to known Na-Th phosphate, which has a non-centrosymmetric structure (Cc space group). The packing of cerium and phosphate groups forms a tree-dimension framework structure with large channels along the *c*-axis, containing disordered sodium atoms. 

Recently, we demonstrated that a solid phase with structural and morphological characteristics analogous to NaCe_2_(PO_4_)_3_(nano) can form from X-ray amorphous ThO_2_ under hydrothermal conditions at pH = 4.8 in a 1 M sodium–phosphate buffer [41]. Interestingly, when the hydrothermal treatment of amorphous ThO_2_ was conducted at pH ~8, the resulting product was a less crystalline analogue of NaTh_2_(PO_4_)_3_. In contrast, CeO_2_ transforms into a well-crystallised double sodium–cerium phosphate phase at pH = 7.7. Taken together, these findings suggest the greater kinetic stability of cerium in phosphate matrices under near-neutral pH conditions compared to thorium.

**Figure 2:**
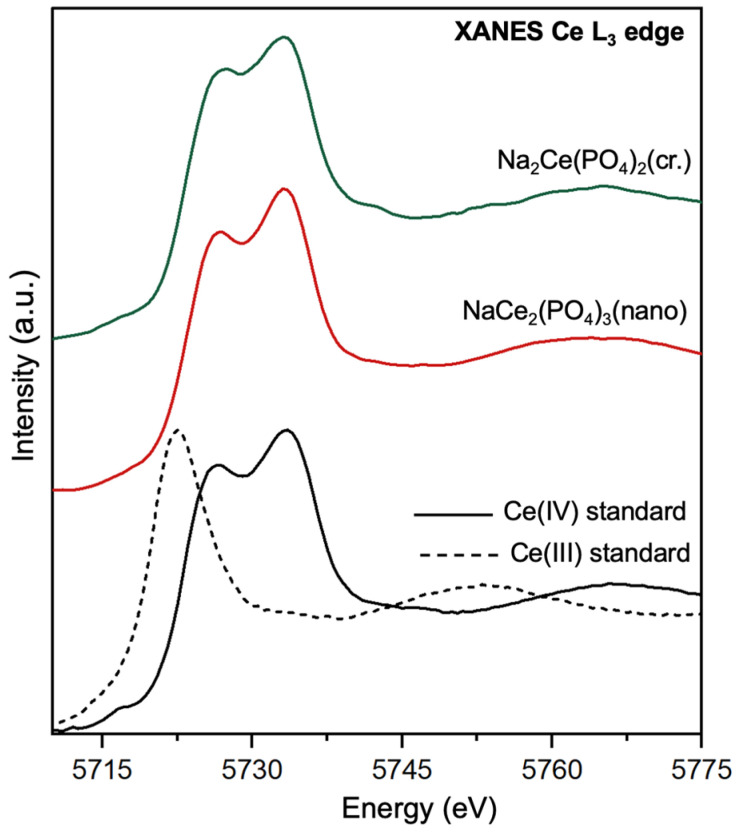
XANES spectra at the Ce L_3_ edge for Na_2_Ce(PO_4_)_2_(cr.) and NaCe_2_(PO_4_)_3_(nano) compared with reference compounds. The spectral features of both synthesised phosphates closely resemble those of the Ce(IV) standard, indicating that cerium is predominantly in the tetravalent oxidation state. Crystalline Ce(OH)PO_4_ and CePO_4_ were used as references for Ce(IV) and Ce(III).

As cerium is a redox-sensitive element, XANES spectroscopy was employed to confirm the predominant oxidation state of Ce in the synthesised phosphate samples. Figure 2 presents the XANES spectra near the Ce L_3_ edge for both the nanocrystalline and crystalline sodium–cerium phosphate phases, alongside reference spectra of Ce(III) and Ce(IV) standards. The Ce L_3_ edge XANES spectra for various cerium compounds reveal features associated with their oxidation states [42]. Ce(IV) compounds exhibit a double-peaked profile, which corresponds to transitions from the 4f^0^ configuration to final 4f^1^5d^1^ states. The second peak after the absorption edge (around 5738 eV) in Ce^IV^ spectra is associated with multielectron excitations [42,43,44]. In contrast, the spectrum of CePO_4_, representing Ce^III^ with a 4f^2^5d^1^ configuration, shows a single main peak at a lower energy. The oxidation state is typically identified via analysis of the position and the shape of the white line. The Ce L3 edge features of synthesised double Na-Ce phosphates match with the spectra of the Ce(IV) standard and confirm the dominance of tetravalent cerium in the structure. This observation is further supported by the first derivative of the XANES spectra, which reveals a clear alignment between the absorption maxima of the synthesised phosphate samples and the Ce^(IV)^(OH)PO_4_ reference compound (Figure S2). The difference in the intensity of the main edge peaks of Na_2_Ce(PO_4_)_2_(cr.) and NaCe_2_(PO4)_3_(nano) could be attributed to changes in the phosphate crystal structure, variations in the local coordination environment, and the redistribution of 5d states. The sensitivity of the Ce L_3_ main edge intensity to structural changes is well documented for oxide, sulphate, and phosphate structures [42,45,46].

### Na-Ce Phosphate Dissolution

**Figure 3:**
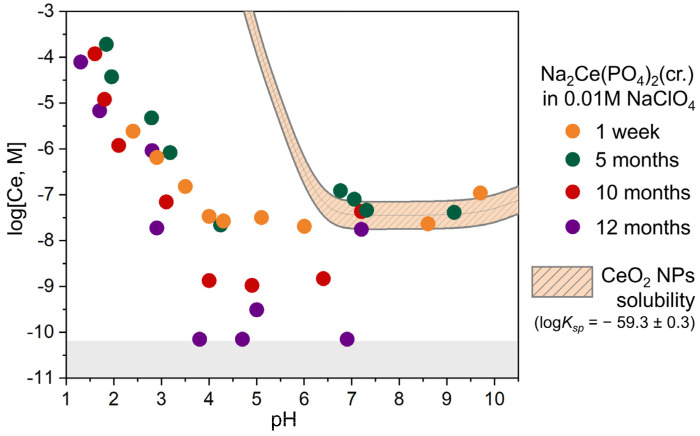
Dissolved cerium concentrations in the presence of Na_2_Ce(PO_4_)_2_(cr.) as a function of the pH at different dissolution times in 0.01 M NaClO_4_. The orange cross-hatched area shows the model of nanosized CeO_2_ solubility, calculated using log*K_sp_* = −59.3 ± 0.3. ‘NPs’ refers to nanoparticles. The grey area denotes the analytical detection limit of cerium concentration measurements using ICP-MS.

To evaluate the phase composition stability of the synthesised sodium–cerium phosphates under varying pH conditions, Na_2_Ce(PO_4_)_2_(cr.) and NaCe_2_(PO_4_)_3_(nano) were stored in aqueous solutions (I = 0.01 M) for up to one year at 25 °C. The dependence of the dissolved cerium concentration in the presence of Na_2_Ce(PO_4_)_2_(cr.) as a function of the pH is presented in Figure 3. The dissolution profile can be divided into two distinct regions. In the acidic-to-near-neutral region (1.5 < pH < 7), the cerium concentration decreases significantly with increasing pH, from approximately 3 × 10^−5^ M to around 3 × 10^−10^ M (near the detection limit). Additionally, a time-dependent decrease in dissolved cerium concentration can be observed, indicating continued solid–solution interaction and a slow approach to equilibrium. At pH values above 7, the measured cerium concentration increases and stabilises around 10^−7^ M, with no noticeable dependence on dissolution time.

The phosphorus concentration, measured using ICP-MS alongside cerium, follows a similar pH-dependent trend in the acidic range (1.5 < pH < 7), decreasing in parallel with cerium (Figure S3). Interestingly, the absolute phosphorus concentrations exceed those that would be predicted from the stoichiometry of the Na_2_Ce(PO_4_)_2_(cr.) compound, which can likely be attributed to the surface sorption of loosely bound phosphate groups that are difficult to fully remove during routine post-synthesis washing. More notably, at pH > 7, the phosphorus concentrations in the solution become irregular and show no clear correlation with the cerium concentration, suggesting possible changes in the solubility-controlling phase under alkaline conditions.

Currently, reliable thermodynamic data for cerium phosphates are limited to trivalent cerium phosphate CePO_4_. The solubility curve for CePO_4_ was calculated using a solubility product of log*K_sp_* = −26.2 and compared with experimental data for Na_2_Ce(PO_4_)_2_(cr.) (Figure S4). Overall, the calculated and experimental pH-dependent dissolved cerium concentrations are in good agreement and exhibit a characteristic V-shaped curve, with the minimum cerium concentration observed at pH 6–7. The experimentally measured cerium concentrations at pH < 7 for Na_2_Ce(PO_4_)_2_(cr.) are slightly lower than the calculated values, which is consistent with the literature reports indicating that tetravalent phosphates generally exhibit lower solubility compared to their trivalent homologues. At pH > 7, notable discrepancies between the calculated and experimental solubilities become apparent.

During the dissolution experiments, both the pH and redox potential (Eh) of the suspensions of Na-Ce(IV) phosphate solids in 0.01 M NaClO_4_ solution were monitored. A comparison of the experimental redox conditions with the Pourbaix diagram for the Ce^4+^/Ce^3+^–PO_4_^3−^–H_2_O system shows that, with increasing pH, the system approaches the area of CeO_2_ stability, indicating the possibility of the formation of CeO_2_ under alkaline conditions (Figure S5). 

Figure 3 compares the experimental dissolution data for Na_2_Ce(PO_4_)_2_(cr.) with the calculated solubility profile of nanosized CeO_2_, based on the equilibria listed in Table S1 and a literature-derived solubility product log*K_sp_* = −59.3. This thermodynamic constant describes the solubility of CeO_2_ nanoparticles with a size range of 2–5 nm [28,46]. Notably, the calculated cerium concentrations corresponding to CeO_2_ solubility closely match the experimental values measured at pH > 7 during the dissolution of Na_2_Ce(PO_4_)_2_(cr.). It is suggested that CeO_2_ may form under alkaline conditions through a dissolution/precipitation mechanism driven by their high thermodynamic oxide stability compared to Na_2_Ce(PO_4_)_2_(cr.).

**Figure 4:**
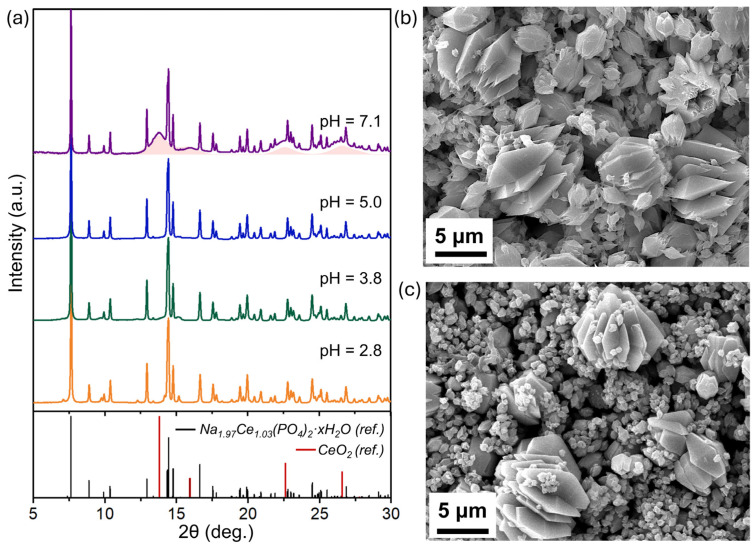
(**a**) XRD patterns of Na_2_Ce(PO_4_)_2_(cr.) samples after 11 months of equilibration in aqueous solution at various pH values (2.8–7.1). The reference patterns for Na_1.97_Ce_1.03_(PO_4_)_2_·xH_2_O [34] and CeO_2_ [PDF 81-792] are shown for comparison. SEM images of the solid phase after dissolution at (**b**) pH = 5.0 and (**c**) pH = 7.1.

To reveal the structural transformations during dissolution, Na_2_Ce(PO_4_)_2_(cr.) solids were collected after 11 months of storage in solution at different pHs and then analysed using XRD, SEM, and Raman spectroscopy. XRD analysis showed that at pH 2.8, the initial crystalline phase of Na-Ce(IV) phosphate dominates, with minor impurities arising from recrystallisation products (Figure 4a). At pH 3.8 and 5.0, the primary phase remains stable, reflecting good structural preservation under moderately acidic to neutral conditions. At pH 7.1, in addition to the well-defined narrow diffraction peaks corresponding to Na_2_Ce(PO_4_)_2_(cr.), broad diffraction maxima can also be observed (Figure 4a). Their positions coincide with those characteristic of fluorite-type CeO_2_, and the significant FWHM values indicate the formation of nanocrystalline CeO_2_ particles. The crystallite size of the secondarily formed CeO_2_ nanoparticles, calculated from the FWHM of (111) and (200) diffraction lines using the Scherrer equation, is established as 2.6 ± 0.3 nm.

SEM images of the Na_2_Ce(PO_4_)_2_(cr.) stored in solution at pH 5.0 for 11 months reveal two distinct particle populations: smaller particles with an average size of approximately 2 μm and a morphology similar to the initial material and larger crystallites measuring up to 6 μm (Figure 4b). As no additional crystalline phases were detected via XRD in these samples, it is reasonable to assume that all observed crystallites are of the Na_2_Ce(PO_4_)_2_(cr.) phase. The larger crystallites are likely secondary Na_2_Ce(PO_4_)_2_(cr.) that formed through the coarsening of the original particles during the ageing process. Such crystallisation may also account for the decrease in dissolved cerium concentration at a near-neutral pH over time.

Particle coarsening and the presence of two distinct size-derived generations (1 μm and 6 μm on average) of the Na_2_Ce(PO_4_)_2_(cr.) phase can also be observed after storage and dissolving in solution at pH 7.1 (Figure 4c). However, CeO_2_ nanoparticles, clearly detected via XRD in this sample, are not observed in the SEM images. This is likely due to the localised nature of the SEM technique and the difficulty in distinguishing nanoscale oxide crystallites from the larger, well-formed phosphate particles. Therefore, Raman spectroscopy measurements were performed to indicate the presence of CeO_2_ nanoparticles after Na_2_Ce(PO_4_)_2_(cr.) dissolution at pH > 7 to further support the XRD data.

**Figure 5:**
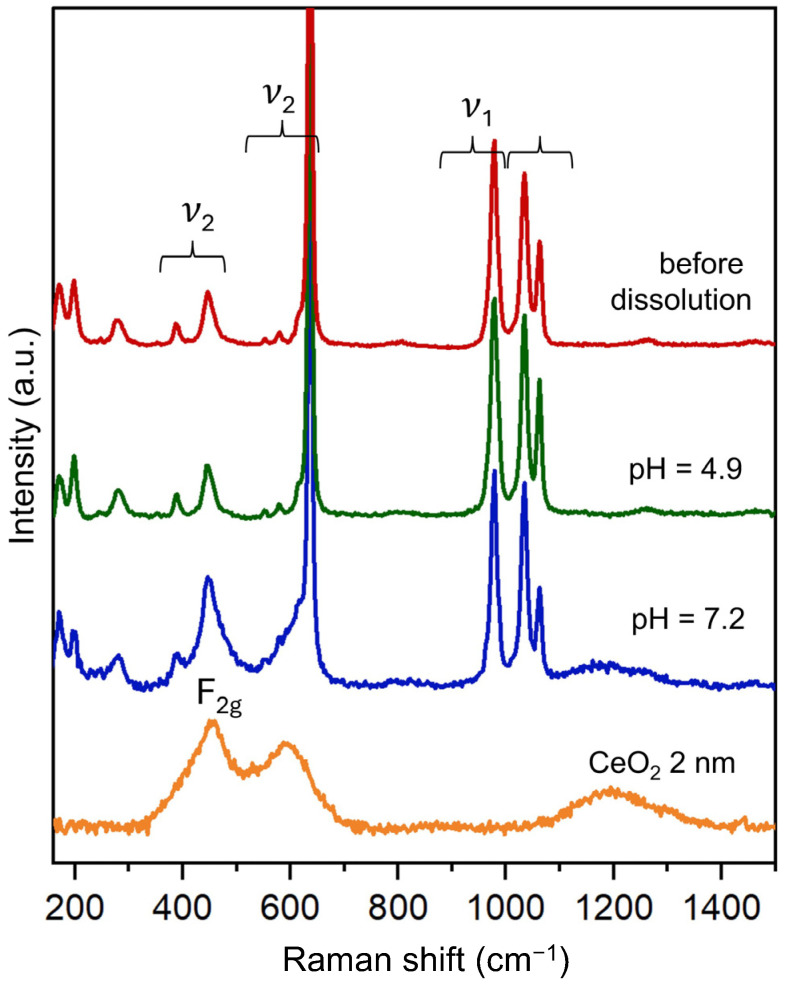
Raman spectra of the Na_2_Ce(PO_4_)_2_(cr.) samples before and after long-term dissolution at pH 4.9 and 7.2, compared with a reference spectrum of CeO_2_ nanoparticles (2 nm). Spectra obtained at a laser wavelength of 405 nm. The symbols ν_1_, ν_3_, and ν_2_ denote different stretching and bending modes of the PO_4_^3−^ group in double Ce(IV) orthophosphate and F_2g_ to the vibrational mode of the oxygen in the CeO_2_ structure.

The Raman spectra of all the analysed Na-Ce(IV) phosphate samples exhibit characteristic vibrational modes corresponding to orthophosphate groups (Figure 5). The Raman modes observed in the range of 1022–1100 cm^−1^ and 977 cm^−1^ can be assigned to the asymmetric (ν_3_) and asymmetric (ν_1_) stretching of the PO_4_^3−^ group. Similarly, the modes observed in the intermediate range of 370–480 cm^−1^ and 553–650 cm^−1^ are the symmetric and asymmetric bending modes of the PO_4_^3−^ group (ν_2_). The characteristic features are consistent with previously reported data for monoclinic double K-Ce(IV) orthophosphate and indicate the absence of polyphosphate species in the structure [37,39].

The spectrum collected after equilibration at pH 7.2, in addition to the phosphate band, displays a broad band centred around 455 cm^−1^ and 590 cm^−1^. This feature is absent from the Raman spectrum of the initial Na-Ce(IV) phosphate sample and those stored at pH 4.9, but it matches well with the vibration modes observed for 2 nm CeO_2_ nanoparticles. The Raman spectrum of CeO_2_ 2 nm nanoparticles recorded under 405 nm excitation is shown along with Na_2_Ce(PO_4_)_2_(cr.). It exhibits the characteristic F_2g_ mode at ~455 cm^−1^, corresponding to the symmetric vibrational mode of the oxygen atoms around cerium ions (O–Ce–O) in agreement with the literature [47,48]. Additionally, a broad feature around 570 cm^−1^ is observed, which could be associated with defect-induced modes or second-order phonon processes in nanocrystalline CeO_2_ [49]. This band typically becomes more pronounced under specific excitation conditions, particularly UV or near-UV Raman, and is often absent or weak in conventional visible Raman spectra. Altogether, the Raman data indicate that after long-term dissolution at pH 7.1, the sample contains a mixture of the original Na_2_Ce(PO_4_)_2_ phase and newly formed CeO_2_ nanoparticles. These findings fully agree with the XRD results, which also reveal broad diffraction features consistent with fluorite-type CeO_2_.

**Figure 6:**
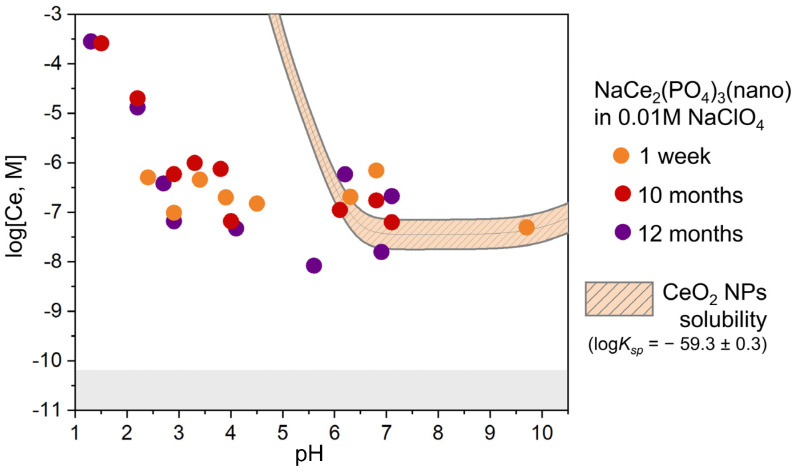
Dissolved cerium concentrations (log[Ce], M) in the presence of NaCe_2_(PO_4_)_3_(nano) as a function of the pH at different dissolution times in 0.01 M NaClO_4_. The orange cross-hatched area shows the model of nanosized CeO_2_ solubility, calculated using log*K_sp_* = −59.3 ± 0.3. ‘NPs’ refers to nanoparticles. The grey area denotes the analytical detection limit of cerium concentration measurements using ICP-MS.

The dependence of the cerium concentration in solution in the presence of NaCe_2_(PO_4_)_3_(nano) on pH values is presented in Figure 6. The dissolution behaviour of NaCe_2_(PO_4_)_3_(nano) in 0.01 M NaClO_4_ reveals notable differences compared to the previously discussed Na_2_Ce(PO_4_)_2_(cr.) phase. In the acidic pH range (pH 1–6), the cerium concentration in solution decreases from approximately 5 × 10^−4^ M to 1 × 10^−8^ M. Importantly, no systematic variation in cerium concentration is observed with increasing dissolution time (1 week, 10 months, and 12 months), suggesting that the solid phase remains structurally and chemically stable during dissolution. This contrasts with the behaviour of Na_2_Ce(PO_4_)_2_(cr.), where dissolution is accompanied by particle recrystallisation and structural evolution. At near-neutral and alkaline pH, the measured cerium concentrations align well with the calculated solubility of nanosized CeO_2_, shown as the cross-hatched area in Figure 6. This correspondence suggests that, as in the case of the dissolution of Na_2_Ce(PO_4_)_2_(cr.), a secondary phase of CeO_2_ nanoparticles may also form under these conditions.

**Figure 7:**
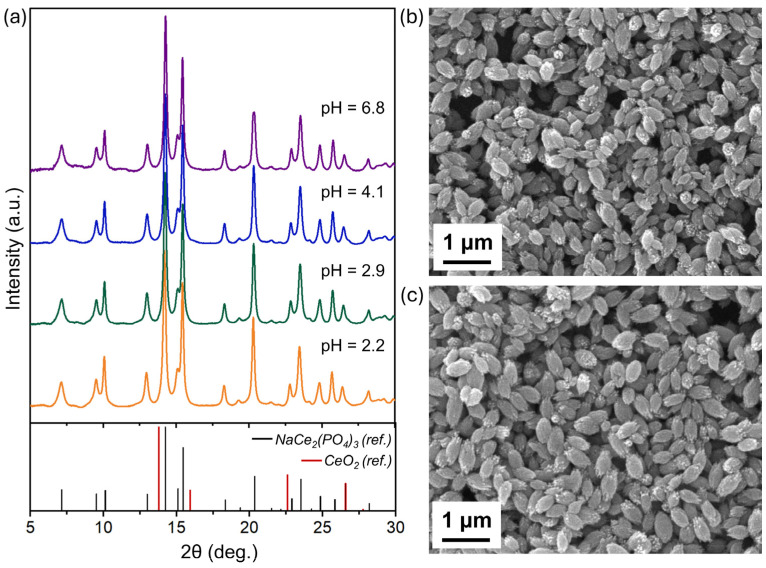
(**a**) XRD patterns of NaCe_2_(PO_4_)_3_(nano) samples after one year of equilibration in aqueous solution at various pH values (2.8–7.1). The reference patterns for NaCe_2_(PO_4_)_3_ [29] and CeO_2_ [PDF 81-792] are shown for comparison [29]. SEM images of the solid phase after dissolution at (**b**) pH 2.9 and (**c**) pH 6.8.

As in the case of Na_2_Ce(PO_4_)_2_(cr.), the solid phase of NaCe_2_(PO_4_)_3_(nano) was comprehensively analysed after long-term equilibration in solution under different pHs using XRD, SEM, and Raman spectroscopy. The XRD patterns (Figure 7a) show no detectable changes in the crystalline phase across the studied pH range. The diffraction peaks remain broad throughout, indicating the nanocrystalline character of NaCe_2_(PO_4_)_3_(nano) even after prolonged dissolution. This behaviour contrasts with the case of Na_2_Ce(PO_4_)_2_(cr.), where additional crystallisation during ageing is observed. Moreover, no additional diffraction peaks attributable to secondary crystalline phases are detected at pH ~3, whereas in the crystalline Na-Ce phosphate system, such conditions lead to the formation of new crystalline precipitates.

SEM imaging reveals no appreciable morphological changes after dissolution (Figure 7b,c). At pH 2.9, the mean aggregate length and width are measured as 419.7 nm and 261.9 nm, respectively, while at pH 6.8, the corresponding values are 471.1 nm and 256.4 nm. These dimensions agree with the original particle size within the experimental uncertainty. Overall, these XRD and SEM results confirm the high stability of the NaCe_2_(PO_4_)_3_(nano) phase and the preservation of its morphological characteristics under acidic-to-near-neutral conditions.

Attempts to identify the formation of CeO_2_ nanoparticles following the dissolution of NaCe_2_(PO_4_)_3_(nano) at pH > 7 were limited by the low buffering capacity of aqueous media in the near-neutral range, and reliable solid-phase characterisation was only possible for the sample collected at pH = 6.8. Under these conditions, neither XRD (Figure 7a) nor Raman spectroscopy (Figure S6) provided conclusive evidence for the presence of CeO_2_. Nonetheless, the possibility of secondary CeO_2_ nanoparticle formation at higher pH values cannot be entirely excluded, as thermodynamic predictions suggest its increased stability under alkaline conditions. Importantly, under similar pH conditions and over the same ageing period (almost one year), CeO_2_ nanoparticle formation is clearly observed for the crystalline Na_2_Ce(PO_4_)_2_ phase, but not for the nanophosphate NaCe_2_(PO_4_)_3_. This strongly suggests that the extent of CeO_2_ formation during the dissolution of the nanophosphate is significantly low.

## 3. Discussion

In this study, we report for the first time a systematic investigation into the dissolution behaviour of two tetravalent cerium phosphate phases: the crystalline double phosphate Na_2_Ce(PO_4_)_2_(cr.) and the nanocrystalline phase NaCe_2_(PO_4_)_3_(nano). The dissolution data, combined with structural and morphological analyses of the solids, reveal distinct differences in their long-term stability and phase evolution across a wide pH range.

For Na_2_Ce(PO_4_)_2_(cr.), our results demonstrate clear pH-dependent transformations in solution. At a low pH, the material undergoes partial reprecipitation and phase reorganisation, while at a near-neutral pH, the original structure is retained but undergoes crystallisation and coarsening, as evidenced in the SEM data. Under alkaline conditions (pH > 7), the dissolution data, XRD, and Raman spectroscopy suggest the formation of CeO_2_ nanoparticles. Notably, Fourest et al. investigated the dissolution of the pure thorium phosphate-diphosphate Th_4_(PO_4_)_4_P_2_O_7_, which was measured in 0.1 M NaClO_4_. The results show that the total concentration of thorium in solution is mainly controlled by the precipitation of two compounds: thorium bis(hydrogen phosphate) in acidic media (pH < 4.5) and thorium hydrated oxide in basic and near-neutral media [13]. Our findings for Na_2_Ce(PO_4_)_2_(cr.) exhibit a broadly comparable dissolution behaviour, with a decrease in cerium concentration in acidic media and a plateau or increase at a near-neutral pH, leading to CeO_2_ nanoparticle formation. 

In contrast, the nanocrystalline phase NaCe_2_(PO_4_)_3_(nano) exhibits a higher cerium concentration in solution than the crystalline double Na-Ce(IV) phosphate under near-neutral conditions. At the same time, no evidence of recrystallisation, structural decomposition, or the formation of secondary phases was observed, even after the long-term contact of nano double Ce(IV) phosphate with an aqueous solution. The retention of its low crystallinity and morphology suggests that this phase is highly kinetically stable. This observation, together with the formation of CeO_2_ nanoparticles as a result of dissolution Na_2_Ce(PO_4_)_2_(cr.) at pH > 7, is particularly significant in light of recent studies highlighting the unusual stability of certain materials in nanocrystalline form, contrary to classical expectations of higher solubility and reactivity in nanoscale solids.

The remarkable stability of the nanocrystalline NaCe_2_(PO_4_)_3_ phase observed in this study aligns with a growing body of evidence suggesting that certain materials exhibit unexpectedly high thermodynamic solubility at the nanoscale. Notably, Navrotsky et al. [50] demonstrated that certain iron oxide polymorphs, such as goethite and maghemite, become thermodynamically stabilised at the nanoscale due to surface energy effects. Their study showed that polymorphs metastable in bulk form can become energetically favoured as nanoparticles, with hydration playing a key role in reducing surface enthalpy [50,51,52,53,54]. Our previous work has demonstrated that the hydrated surfaces of CeO_2_ nanoparticles significantly reduce the surface energy and promote the long-term stability of 2 nm nanoparticles even across a 4.5-year dissolution timeline [46].

Similarly to oxide-based systems, the exceptional phase stability of nanocrystalline NaCe_2_(PO_4_)_3_ may be attributed to structural features such as hydrated sites, partially associated with sodium within the channels, as demonstrated for the analogous NaTh_2_(PO_4_)_3_ system [41]. This interpretation is supported by TGA data, which indicate a higher hydration of the nanophase compared to crystalline Na_2_Ce(PO_4_)_2_ (Figure S1). On the other hand, the formation of CeO_2_ as result of the dissolution of the Na_2_Ce(PO_4_)_2_(cr.) phase may be linked to its dissolution mechanism. While nanophase cerium phosphate likely dissolves congruently, the crystalline version appears to undergo incongruent dissolution. A similar phenomenon was reported for crystalline monazite (CePO_4_), where partial dissolution led to CeO_2_ formation, while the phosphate framework remained largely intact [55]. This highlights the importance of considering both structural hydration and dissolution mechanisms when evaluating the stability of Ce-phosphate systems.

In our recent work, we demonstrated that the nanocrystalline phase NaCe_2_(PO_4_)_3_(nano) can form spontaneously from CeO_2_ nanoparticles under prolonged exposure to phosphate-containing solutions [29]. The transformation was strongly pH-dependent: at pH ~4, CeO_2_ underwent structural changes, while at pH ~8, the oxide nanoparticles remained largely unaltered even after extended contact. However, the driving force behind this transformation remained unclear. In this study, we show that NaCe_2_(PO_4_)_3_(nano) exhibits markedly reduced cerium release into solution compared to CeO_2_ under acidic conditions (pH < 6). At higher pH levels, however, the concentrations of dissolved cerium from both phases are comparable. This suggests that the transformation of CeO_2_ into NaCe_2_(PO_4_)_3_(nano) is driven by the lower solubility and greater thermodynamic stability of the phosphate phase under acidic and near-neutral conditions. Both phases may coexist at pH > 6, where the solubilities are close, indicating competitive stability and the potential for equilibrium between oxide and phosphate forms. 

In conclusion, the findings establish key thermodynamic stability conditions for Na–Ce(IV) phosphate and CeO_2_ phases, which is critical in predicting cerium mobility in both natural and engineered systems, including nuclear waste immobilisation and environmental remediation. Given the close similarity in ionic radii and coordination behaviour between Ce(IV), Th(IV), and Pu(IV) (r[Ce^4+^] = 0.97 Å, r[Th^4+^] = 1.05 Å, r[Pu^4+^] = 0.96 Å for coordination number CN = 8 [56]), cerium is commonly used as a non-radioactive surrogate for tetravalent actinides. Several isostructural phosphate compounds have been reported for Ce(IV) and Th(IV), particularly in systems containing potassium, ammonium, and recently sodium as charge-balancing cations [29,35,40,57,58,59]. Structural data on Pu(IV) phosphates remain scarce due to the radioactivity and strong redox sensitivity of plutonium. However, cerium’s ability to reversibly switch between Ce^3+^ and Ce^4+^ oxidation states makes it especially relevant in modelling systems where Pu(IV)/Pu(III) redox transitions play a role under reducing conditions. These chemical analogies reinforce the validity of cerium-based phosphates as surrogate systems for probing the dissolution dynamic and long-term evolution of actinide-hosting materials in geochemically relevant contexts.

## 4. Materials and Methods

### 4.1. Synthesis of CeO_2_ Nanoparticles

CeO_2_ nanoparticles were synthesised via rapid chemical precipitation from a Ce(IV) solution using aqueous ammonia. A 0.1 M aqueous solution of (NH_4_)_2_[Ce(NO_3_)_6_] was prepared by dissolving 2.74 g of the solid salt in 50 mL of water. Under continuous stirring, 200 mL of deionised water and 50 mL of concentrated aqueous ammonia were mixed, then the prepared (NH_4_)_2_[Ce(NO_3_)_6_] solution was added dropwise to the ammonia solution. Stirring was maintained for 2 h, resulting in the formation of a light-yellow precipitate of CeO_2_ nanoparticles. The resulting precipitate was washed three times with MilliQ water to remove synthesis by-products, using centrifugation for phase separation. The particle size distributions obtained from HRTEM data revealed relatively low sample polydispersity, with an average nanoparticle size of 2.4 nm.

### 4.2. Hydrothermal Treatment

The as-prepared CeO_2_ nanoparticles were subjected to hydrothermal treatment in sodium phosphate-buffer solutions. The buffers were prepared by mixing 1M Na_2_HPO_4_ and 1M NaH_2_PO_4_ solutions. The first buffer solution had a Na_2_HPO_4_/NaH_2_PO_4_ volume ratio of 1:12 and a pH of 4.4 after equilibration under air. The second buffer had a Na_2_HPO_4_/NaH_2_PO_4_ volume ratio of 14:1 and a final pH of 7.7. The CeO_2_ nanoparticle suspensions in the phosphate buffers were placed in Teflon-lined autoclaves and heated at 200 °C in a drying oven for 8 h. After treatment, the samples were rinsed thoroughly with MilliQ water.

### 4.3. Dissolution Experiments

Dissolution experiments were performed under undersaturation conditions [28,60]. For this, double cerium–sodium phosphates in the form of concentrated aqueous suspension were deposited into polypropylene tubes. The pH values were adjusted in the range of 1.5–10 via the successive addition of dilute solutions of NaOH and HClO_4_. The overall solid concentration in the samples was 70–100 mg/L for samples at pH < 4 and 30–40 mg/L for samples at pH > 4. Dissolution studies were performed at a constant ionic strength (0.01 M NaClO_4_) at 25.0 ± 0.5 °C. The pH values of the resulting suspensions were determined using an InLab Expert Pro pH electrode (Mettler Toledo, Columbus, OH, USA). The redox potential (E(mV)) was measured using a Pt electrode relative to a Ag/AgCl reference electrode and converted to Eh through the following equation: Eh (mV) = E (mV) + 223.054 − 0.6985∙t (t, the temperature of the sample liquid). Zobell’s standard solution was used for the control.

The pH and the concentrations of cerium and phosphorus in the solutions were monitored throughout the experiments. Sampling was performed within 1 week to 12 months of equilibration. The elemental concentrations in the solution were determined via ICP-MS.

### 4.4. Inductively Coupled Plasma Mass Spectrometry (ICP-MS) Measurements 

The quantitative determination of ^31^P and ^140^Ce was performed using ICP-MS with the PlasmaQuant MS Elite (Analytic Jena, Jena, Germany). All samples were diluted with the 1% nitric acid solution to concentrations within the calibration range. The dilution solution was prepared using ultra-high-purity HNO_3_ and deionized water obtained using a Milli-Q water purification system (resistivity, 18.2 MΩ·cm). The calibration range was 100–1000 µg/L and 0.1–100 µg/L for ^31^P and ^140^Ce, respectively. For signal correction and matrix effect compensation, an internal standard with 103Rh and 193Ir was used. The detection limit for cerium measurements via ICP-MS was 0.01 μg/L (7 × 10^−11^ M). Each data point represents the average of three independent measurements (*n* = 3), with 10 scans per measurement and 10 replicates per sample. For each measurement, the relative standard deviation was within 10%, corresponding to an uncertainty of approximately ±0.1 in the logarithmic value of the Ce concentration.

### 4.5. Thermodynamic Modelling

The PHREEQC code [61] was employed to model CeO_2_ solubility under the experimental redox conditions. The corresponding Eh values used in the calculations are provided in Figure S5. The set of equilibrium reactions and thermodynamic constants used for the modelling is listed in Table S2.

### 4.6. Characterisation Methods

Synchrotron-based XRD measurements were performed using the X-ray structural analysis beamline of the Kurchatov Synchrotron Radiation Source (NRC «Kurchatov Institute» Moscow) [62]. For synchrotron-based XRD measurements, Na-Ce(IV) phosphate samples were placed in synthetic vacuum oil and mounted on a 200 nm nylon CryoLoop. The measurements were performed in the transmission mode, using a Rayonix SX-165 CCD detector (Rayonix, LLC, Evanston, IL, USA) at a wavelength of 0.75 Å. Raw 2D scattering images were integrated using the Fit2D software (ver. 18 beta). The PDF4+ database was used to identify the crystalline phases.

X-ray absorption near-edge structure (XANES) measurements at the Ce L_3_ edge were performed using an X-ray laboratory spectrometer located at the Department of Radiochemistry, Moscow State University (Moscow, Russia) [63]. The spectrometer operates based on Johann geometry and is equipped with an X-ray tube with a silver anode and a silicon drift detector (FASTSDD; Amptek Inc., Bedford, MA, USA). A curved Ge crystal monochromator was used for monochromatizing and focusing the X-ray beam, allowing energy scanning in the range of 5710–5780 eV for cerium (reflection 3 3 3). A helium-filled chamber was utilised along the entire optical path of the X-ray beam to prevent signal loss due to air absorption when measuring the Ce L_3_-edge spectrum. All measurements were conducted at room temperature. The samples were sealed between two 25 μm thick layers of Kapton. Data were collected in transmission mode, with each spectrum representing the sum of 15 scans, which were merged and normalised using the IFEFFIT software package (ver. 0.9.26) [64]. The spectra of well-characterised crystalline Ce(OH)PO_4_ and CePO_4_ were used as references for Ce(IV) and Ce(III) [34]. XANES measurements were not performed on solution samples due to the extremely low cerium concentrations, which were below the detection limit for this technique.

The sample morphology was studied using a JSM-IT500 SEM (JEOL Ltd., Tokyo, Japan) with a tungsten thermionic cathode and equipped with an Oxford X-Max-*n* EDS instrument. Samples deposited on a silicon substrate were covered with a 20–25 nm thick conductive carbon film using a vacuum evaporator. For energy-dispersive X-ray (EDX) analysis, the electron probe current was set at 0.7 nA, and the exposure time was 60 s. The detection thresholds for all analysed elements reached 0.1–0.2 wt%. The obtained spectra were processed using INCA (version 21b) software using the XPP correction model. 

Raman spectra were obtained using a Renishaw inVia Raman spectrometer with a 50 mW laser diode at a wavelength of 405 nm. The spectral range was set between 100 and 2000 cm^−1^. Laser light was focused on the sample through a 50× objective to a spot size of ~2 μm. The power on the sample was <0.1 mW.

## Data Availability

Dataset available on request from the authors.

## Supplementary Materials

The following supporting information can be downloaded at: https://www.mdpi.com/article/10.3390/molecules30102105/s1, Table S1: Concentration of elements (in atomic percentages) in Na_2_Ce(PO_4_)_2_(cr.); Figure S1: Thermogravimetric analysis (TGA) of Na_2_Ce(PO_4_)_2_(cr.) and NaCe_2_(PO_4_)_3_ (nano); Figure S2: First derivative of the XANES spectra; Figure S3: Phosphorus concentration in solution in presence of (a) Na_2_Ce(PO_4_)_2_(cr.) and (b) NaCe_2_(PO_4_)_3_(nano) at different pHs; Figure S4: The dependence of cerium concentration in solution on pH for Na_2_Ce(PO_4_)_2_(cr.) sample and the calculated solubility of CePO_4_; Figure S5: Pourbaix diagram calculated by Hydra/Medusa software (ver. 18 Aug. 2009) with pH/Eh experimental values in solubility experiments; Table S2: Chemical equilibria and corresponding thermodynamic constants used in this work for modelling the solubility of CeO_2_; Figure S6: Raman spectra of the NaCe_2_(PO_4_)_3_(nano) samples; Figure S7: (a) HRTEM images of CeO_2_ nanoparticles; (b) particle size distributions obtained from HRTEM analysis.

## Abbreviations

The following abbreviations are used in this manuscript:

XRD	X-ray diffraction
SEM	Scanning electron microscopy
ICP-MS	Inductively coupled plasma mass spectrometry
NPs	Nanoparticles
log*K_sp_*	Logarithmic value of the solubility product constant
