# Supplementary material for: Comprehensive Dissolution Study on Two Double Ce(IV) Phosphates with Evidence of Secondary CeO2 Nanoparticle Formation
Source: Molecules. 2025 May 9;30(10):2105. doi: 10.3390/molecules30102105 (PMC12114321; doi:10.3390/molecules30102105)
Supplement: Supplementary file 1 [file molecules-30-02105-s001.zip › molecules-3605303-supplementary.pdf]

Supplementary Information

Comprehensive Dissolution Study on Two Double Ce(IV) Phosphates with Evidence of Secondary CeO_2 Nanoparticle Formation

*Anastasiia L. Listova [1], Anastasiia S. Kuzenkova [1], Mikhail A. Gerasimov [1], Elizaveta S. Kulikova [2], Roman D. Svetogorov [1,2], Daniil A. Novichkov [1], Alexei A. Averin [3], Vasiliy O. Yapaskurt [4], Anna Yu. Romanchuk [1], Stepan N. Kalmykov [1] and Tatiana V. Plakhova [1,]**

[1] *Faculty of Chemistry, Lomonosov Moscow State University, Leninskie Gory 1, 119991 Moscow, Russia*

[2] *National Research Centre Kurchatov Institute, Akademika Kurchatova pl. 1, 123182 Moscow, Russia*

[3] *Frumkin Institute of Physical Chemistry and Electrochemistry, Russian Academy of Sciences, Leninsky Pro-spect 31 bld. 4, 119071 Moscow, Russia*

[4] *Faculty of Geology, Lomonosov Moscow State University, Leninskie Gory 1, 119991 Moscow, Russia*

* *Correspondence: plakhovatv@my.msu.ru*

Table S1. The concentration of elements (in atomic percentages) in $Na_2Ce(PO_4)_2$(cr.) measured by EDX with Na standarted by NaCl.

Sample	atomic %		
	Na	P	Ce
$Na_2Ce(PO_4)_2$(cr.)	15.6	15.13	7.89
	16.17	15.09	7.64
	16	15.05	7.77
	16.15	15	7.76
	16.01	14.95	7.89
	15.96	15	7.85
	16.35	15	7.65
	15.91	15.11	7.75
	16.11	15.07	7.7
	15.89	15.04	7.84
	15.73	15.13	7.82
**Mean**	**15.99**	**15.05**	**7.78**

Std. deviation	0.21	0.06	0.09
Max.	16.35	15.13	7.89
Min.	15.6	14.95	7.64

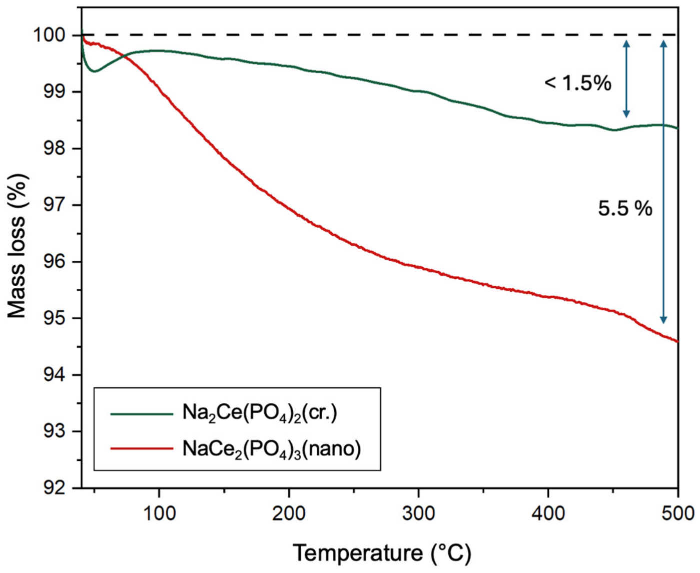

Figure S1. Thermogravimetric analysis (TGA) of $Na_2Ce(PO_4)_2$(cr.) and $NaCe_2(PO_4)_3$ (nano) samples in the temperature range of 25–500 °C. The nanocrystalline phase exhibits a total mass loss of approximately 5.5%, while the crystalline phase shows less than 1.5% mass loss, indicating a higher degree of hydration in the nanomaterial.

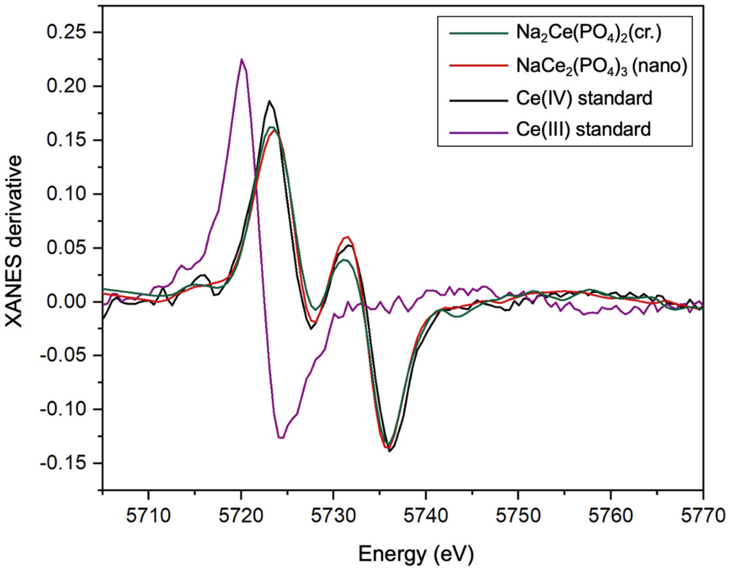

Figure S2. The first derivative of the XANES spectra at the Ce L_3-edge for $Na_2Ce(PO_4)_2$(cr.) and $NaCe_2(PO_4)_3$(nano), compared with Ce(III) and Ce(IV) reference

compounds. The derivative spectra of both phosphate samples closely match that of the Ce(IV) standard, confirming the predominance of Ce(IV) in the synthesised materials. The distinct features of the Ce(III) standard further highlight the absence of a significant Ce^{3+} contribution.

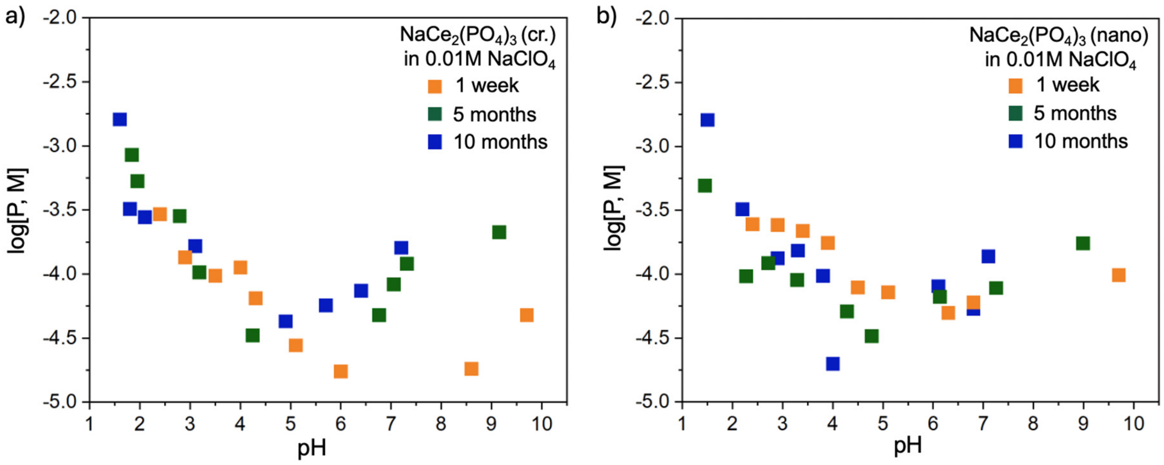

Figure S3. Phosphorus concentration in solution (log[P], M) in the presence of (a) $Na_2Ce(PO_4)_2$(cr.) and (b) $NaCe_2(PO_4)_3$(nano) in 0.01 M $NaClO_4$ at 25 °C over varying time of dissolution (1 week, 5 months, and 10 months).

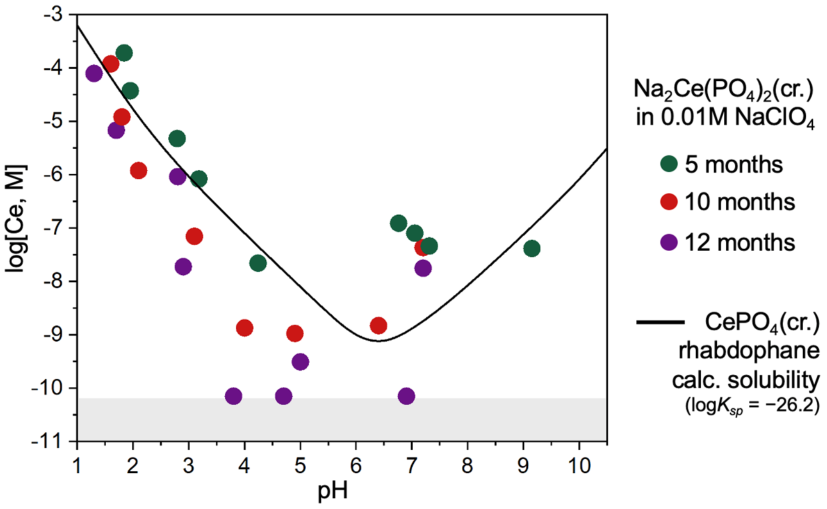

Figure S4. The dependence of cerium concentration (log[Ce], M) in 0.01 M $NaClO_4$ on pH for $Na_2Ce(PO_4)_2$(cr.) sample, measured after different dissolution times. The line represents the calculated solubility of $CePO_4$ with a rhabdophane-type structure based on a known solubility constant (Liu et al., 1997)

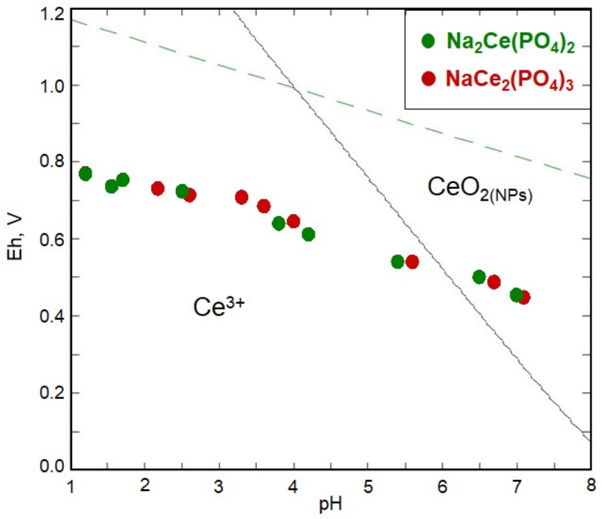

Figure S5. Pourbaix diagram calculated by Hydra/Medusa software with pH/Eh experimental data for $Na_2Ce(PO_4)_2$(cr.) (green circles) and $NaCe_2(PO_4)_3$(nano) (red circles) solubility experiments. $[Ce^{4+}] = 10^{-7}$ M, $[PO_4^{3-}] = 10^{-7}$ M.

Table S2. Chemical equilibria and corresponding thermodynamic constants are used in this work to model the solubility of CeO_2.

Equation	log*K*	Reference
$CeO_2 + e^- + 4H^+ \rightleftharpoons Ce^{3+} + 2H_2O$	25.8 ± 0.3	Plakhova et al., 2016
$CeO_2 + 2H_2O \rightleftharpoons Ce^{4+} + 4OH^-$	− 59.3 ± 0.3	Plakhova et al., 2016
$Ce^{4+} + 5H_2O \rightleftharpoons Ce(OH)_5^- + 5H^+$	−14.6	Estimated at preset work based on the experimental data

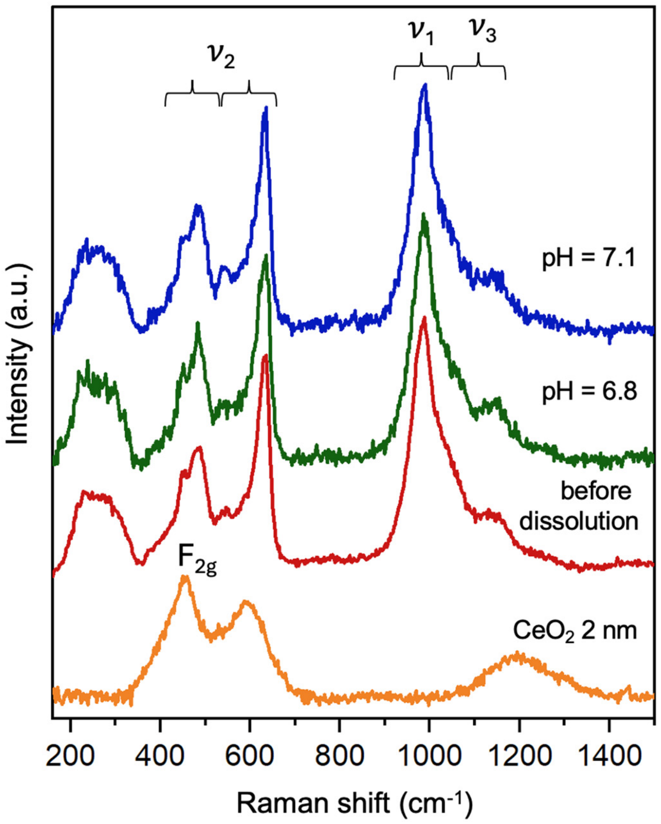

Figure S6. Raman spectra of the $NaCe_2(PO_4)_3$(nano) samples before and after long-term dissolution at pH 6.8 and 7.1, compared with a reference spectrum of CeO_2 NPs (2 nm). Spectra obtained at laser wavelength 405 nm. The symbols ν_1, ν_3, and ν_2 denote different stretching and bending modes of the PO_4^{3-} group in double Ce(IV) orthophosphate and F_{2g} to the vibrational mode of the oxygen in the CeO_2 structure.

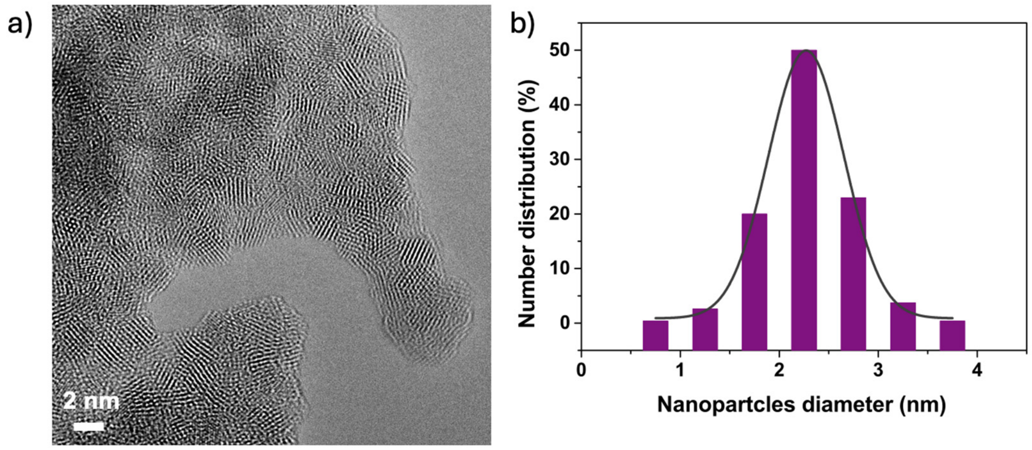

Figure S7. a) HRTEM images of CeO_2 samples synthesised via rapid chemical precipitation from a Ce(IV) solution using aqueous ammonia. b) Particle size distributions obtained from HRTEM analysis.
